# TB or not TB? Development and validation of a clinical decision support system to inform airborne isolation requirements in the evaluation of suspected tuberculosis

**DOI:** 10.1017/ice.2024.214

**Published:** 2025-02

**Authors:** Caitlin M. Dugdale, Kimon C. Zachary, Rebecca L. Craig, Alexandra Doms, Lindsay Germaine, Chloe V. Green, Eren Gulbas, Rocio M. Hurtado, Emily P. Hyle, Michelle S. Jerry, Jacob E. Lazarus, Stephen Maxfield, Molly Paras, Katherine Swanson, Erica S. Shenoy

**Affiliations:** 1 Medical Practice Evaluation Center, Department of Medicine, Massachusetts General Hospital, Boston, MA, USA; 2 Division of Infectious Diseases, Department of Medicine, Massachusetts General Hospital, Boston, MA, USA; 3 Harvard Medical School, Boston, MA, USA; 4 Infection Control, Massachusetts General Hospital, Boston, MA, USA; 5 Department of Medicine, Massachusetts General Hospital, Boston, MA, USA; 6 Clinical Informatics and Decision Support, Digital Health, Mass General Brigham, Somerville, MA, USA; 7 Global Health Committee, Boston, MA, USA; 8 Infection Control, Mass General Brigham, Boston, MA, USA

## Abstract

**Background:**

The study objective was to develop and validate a clinical decision support system (CDSS) to guide clinicians through the diagnostic evaluation of hospitalized individuals with suspected pulmonary tuberculosis (TB) in low-prevalence settings.

**Methods:**

The “TBorNotTB” CDSS was developed using a modified Delphi method. The CDSS assigns points based on epidemiologic risk factors, TB history, symptoms, chest imaging, and sputum/bronchoscopy results. Below a set point threshold, airborne isolation precautions are automatically discontinued; otherwise, additional evaluation, including infection control review, is recommended. The model was validated through retrospective application of the CDSS to all individuals hospitalized in the Mass General Brigham system from July 2016 to December 2022 with culture-confirmed pulmonary TB (cases) and equal numbers of age and date of testing-matched controls with three negative respiratory mycobacterial cultures.

**Results:**

104 individuals with TB (cases) and 104 controls were identified. Prior residence in a highly endemic country, positive interferon release assay, weight loss, absence of symptom resolution with treatment for alternative diagnoses, and findings concerning for TB on chest imaging were significant predictors of TB (all *P* < 0.05). CDSS contents and scoring were refined based on the case–control analysis. The final CDSS demonstrated 100% sensitivity and 27% specificity for TB with an AUC of 0.87.

**Conclusions:**

The TBorNotTB CDSS demonstrated modest specificity and high sensitivity to detect TB even when AFB smears were negative. This CDSS, embedded into the electronic medical record system, could help reduce risks of nosocomial TB transmission, patient-time in airborne isolation, and person-time spent reviewing individuals with suspected TB.

## Introduction

Nosocomial transmission of tuberculosis (TB) gained national attention in the United States during the 1980s in the context of rising incidence, fueled by the surging HIV/AIDS epidemic and weakened public health infrastructure. Incidence of TB declined from 10.4 per 100,000 in 1992 to a nadir of 2.2 per 100,000 in 2020 and nosocomial transmission has been rare in the US in recent years.^1,2^ However, incidence has increased to 2.9 cases per 100,000 in the US and 3.2 per 100,000 in Massachusetts in 2023.^3,4^ At the same time, many hospitals face staffing and capacity challenges, making resource-efficient decision-making for patients with suspected TB more urgent.

Centers for Disease Control and Prevention (CDC) guidelines for the evaluation of persons with suspected pulmonary TB, recommend collecting three sputum samples for acid-fast bacilli (AFB) smear and mycobacterial culture 8–24 hours apart, while implementing airborne infection isolation (AII).^5^ Approximately half of persons with pulmonary TB have negative sputum smears, and cultures usually take at least two weeks to turn positive.^6,7^ Nucleic acid amplification tests (NAAT) for TB have higher sensitivity than AFB smears and have been incorporated into national guidelines for the diagnosis of TB.^7^ However, the sensitivity of NAAT remains significantly lower than that of culture, particularly when the specimen is AFB smear-negative.^8,9^ Therefore, reflexive discontinuation of AII, based on AFB smear and NAAT results alone, may be premature and risk transmission to other patients and to healthcare personnel.

Given the risk of premature discontinuation of AII, healthcare facilities often require additional review of patient records by infection prevention and control (IPC) staff, including infection preventionists (IPs) and physicians, before discontinuation of AII. In addition to reviewing the medical record, IPC staff may discuss the patient’s case with the primary team and/or relevant consultants (*eg*, infectious diseases, pulmonary medicine) to determine whether TB is sufficiently unlikely to warrant discontinuation of AII. These case-by-case determinations are labor- and time intensive.

Clinical decision support systems (CDSS) have been used to improve patient care through supporting clinicians in the implementation of best practices and clinical guidelines. The objective of this study was to develop and validate a CDSS that could be embedded in the electronic health record (EHR) to support discontinuation of AII when appropriate and, for patients determined to be at higher risk of TB, collect and summarize relevant data to support individual evaluation by IPC staff, thereby enabling efficient case review.

## Methods

### Developing the model framework

At Massachusetts General Hospital (MGH), a large academic medical center in the Mass General Brigham (MGB) healthcare system, individuals being evaluated for TB who have negative sputum AFB smears and PCRs are evaluated by IPC staff on a case-by-case basis to determine whether TB is sufficiently improbable to favor AII discontinuation. TB evaluation approaches at MGB have been previously described.^10^ Individuals undergoing evaluation for TB are given a “TB-Risk” infection status in the EHR and AII is maintained. We constructed a CDSS to expedite AII discontinuation while preserving robust sensitivity to maintain AII in the setting of possible AFB- and NAAT-negative TB. We assembled a team of content matter experts, including IPs and infectious disease physicians with extensive experience in IPC and in TB management. Informed by published epidemiologic data and clinical guidelines, questions were drafted, with an associated scoring system initially proposed by the project lead (KCZ). These questions and the associated scoring system were then iteratively revised by Delphi method consensus and by live application of the draft tool toward test cases of selected hospitalized patients evaluated for TB at MGH from January 1, 2023, to June 30, 2024.

### Case–control CDSS validation substudy

To validate the CDSS, we first replicated the CDSS questions and associated scoring system in a REDCap form.^11,12^ We queried the MGB electronic data warehouse, spanning eight MGB acute care facilities in multiple cities,^10^ to identify 104 individuals hospitalized with TB and 104 hospitalized age- and date of test-matched controls with at least three negative mycobacterial cultures from sputum specimens obtained within a 7-day period between July 2016 and December 2022. We then applied the CDSS framework retrospectively to these cases and controls through chart abstraction into REDCap. Clinical characteristics of the 104 hospitalized individuals with TB have been published previously.^10^ We generated summary statistics for all components of the CDSS. We identified potential predictors of TB by calculating unadjusted odds ratios with complete case analysis and using a chi-square test for comparison with p-values <0.05 considered statistically significant. Statistical analyses were conducted in Stata v17 (College Station, TX).

### Refining the model

Variables from the case–control study found to be negatively associated with TB case prediction, or that did not contribute to the predictive ability of the tool, were eliminated from the CDSS or were preserved with downweighted scores. We iteratively assessed the impact of upweighting the scores of predictors of TB case status that were identified in the case–control analysis. With each alteration in the model, we assessed sensitivity, specificity, and area under the curve (AUC) of the CDSS scoring system. We sought to develop a model that demonstrated 100% sensitivity to detect culture-positive TB while also maximizing specificity, maintaining consistency with clinical judgment, and facilitating collection of details relevant to individualized IPC review. We also conducted a subgroup analysis looking at model performance pre-COVID (2016–2019) and post-COVID (2020–2022).

### Estimating the impact on IPC workload

To estimate the potential impact of the tool on IPC person-time at MGH, we multiplied the specificity of the tool (*ie*, the proportion of individuals with suspected TB whose isolation precautions would be correctly, automatically removed by the tool without IPC review) by the number of individuals with suspected TB hospitalized at MGH annually (*ie*, >300). We then applied a conservative estimate of 30 minutes of IPC person-time required for manual review per patient evaluated for TB to estimate overall IPC person-time saved with the tool annually.

### Human subjects

The study was approved by the MGB Institutional Review Board (2012P002359).

## Results

### Achieving Delphi consensus for the initial model

**Figure 1.**
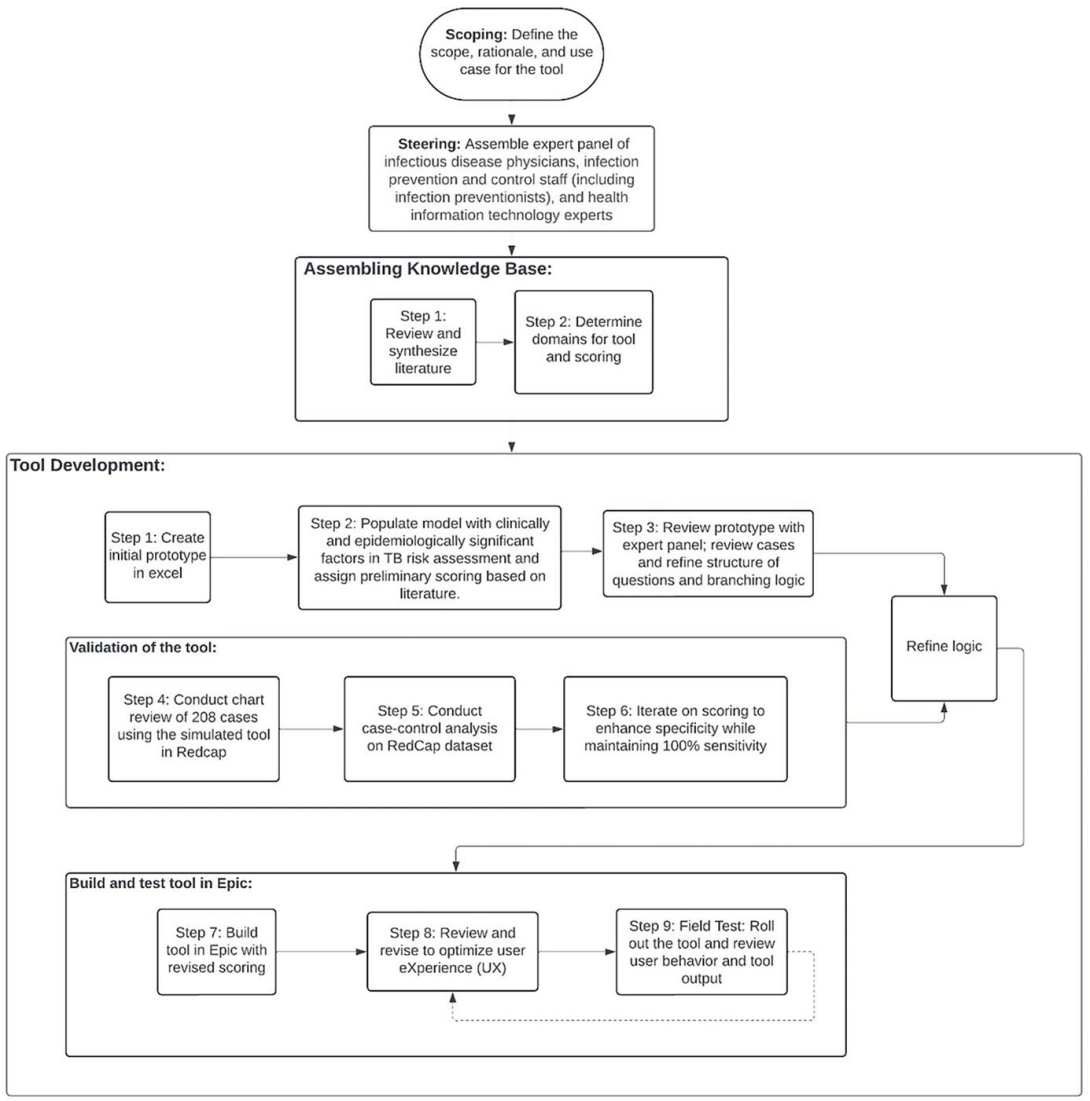
Diagram of the TBorNotTB CDSS development process. Steps in the design, development, and validation of a complex Smartform-based clinical decision support system (CDSS) to guide clinicians through the diagnostic evaluation of hospitalized individuals with suspected pulmonary TB in low prevalence settings.

We drafted a clinical scoring system incorporating epidemiological characteristics, medical conditions known to increase the risk of TB, symptoms, and the results of diagnostic testing, including tuberculin skin tests (TSTs), interferon-gamma release assays (IGRAs), lower respiratory tract specimens (AFB smear and NAAT), and chest imaging (Figure 1, Table 1).^5,7^ Each component of the scoring system (*ie*, chest imaging, epidemiology, history of active or latent TB, past medical history, symptoms, and sputum/bronchoscopy results) was given a minimum and maximum score; scores were summed across components to produce a total score.

**Table 1.** Components of the final (Revised) TBorNotTB CDSS scoring system

SECTION/QUESTION	Max points
**CHEST IMAGING (Score range: -3-10)** ^1,2^	
Cavitary lesion or findings described as suspicious for TB	10
Other findings	3
No lung, pleura, or lymph node abnormalities	-3 CT /-1 CXR
**EPIDEMIOLOGY (Score range: 0-5)^1^**	
Has the patient ever resided outside the US, Canada, Western Europe, Australia, or New Zealand?	5
If no: Has the patient ever traveled outside the US, Canada, Australia, Western Europe, Australia, or New Zealand for more than a month?^2^	1
Has the patient ever been incarcerated?^3^	1
Has the patient ever resided in a homeless shelter?^3^	3
Has the patient ever worked in a correctional facility or homeless shelter?^3^	1
Has the patient ever had a close contact with active TB at any time in their life?	5
**HISTORY OF ACTIVE OR LATENT TB (Score range: 0-5)^1^**	
Has the patient ever been diagnosed with active TB?	5
If yes: Is there documentation of completion of an appropriate treatment regimen?	0
If no: Has the patient ever been diagnosed with latent TB?	5
If yes, Is there documentation of completion of an appropriate treatment regimen?	-2
If no: Has the patient ever had an IGRA or TST?	0
If yes: Has the patient ever had a positive or borderline IGRA or a positive TST (>/= 5 mm of induration)?	3
If no: Has the patient ever had a negative IGRA?	0
If yes: If documented, enter the date of the most recent negative IGRA or TST (points awarded if within the past 30 d).	-1^5^
**PAST MEDICAL HISTORY (Score range: 0)^1^**	
Is the patient moderately to severely immunocompromised?^4^	0
Does the patient have a history of any of the following conditions: HIV, underweight (body mass index <18.5), diabetes mellitus, active head and neck cancer, chronic kidney disease, chronic obstructive pulmonary disease, active tobacco smoking, cirrhosis, silicosis, celiac disease, gastrectomy, or gastric bypass?	0
**SYMPTOMS (Score range: 0-5)^1^**	
Does the patient have any of the following symptoms consistent with active TB: cough, hoarseness, hemoptysis, fever, or night sweats in the past month?	1 each
Does the patient report unplanned weight loss in the past year?	3
Have the symptoms resolved or markedly improved with treatment for an alternative diagnosis, and without any medications active against TB?^6^	-8
**SPUTUM/BRONCHOSCOPY RESULTS (Score range: -9-0)^1^**	
At least three negative AFB smears, at least 8 hours apart	-6
1st negative TB PCR	-3
2nd negative TB PCR	-3
**TOTAL SCORE <1: Workup deemed complete and airborne isolation precautions discontinued** **TOTAL SCORE ≥1: Maintains airborne isolation and advises clinician to continue evaluation for pulmonary TB (** ***eg*** **IGRA, chest CT, or TB PCR) and to route the patient record to individualized review by infection control personnel**	

*TB: TB; IGRA: interferon gamma release assay; AFB: acid fast bacilli; PCR: polymerase chain reaction; CT: computed tomography; CXR: chest radiograph.*

1 The score range reflects the absolute minimum and absolute maximum points allowed for the section; scores outside of this range were brought to their nearest bound for the calculation of total score.

2 The CDSS requires some form of chest imaging in order to complete the form. Results of chest computed tomography (CT) are prioritized over chest radiography.

3 There is a maximum cumulative score of 3 points for these particular questions regarding epidemiology.

4 Conditions that meet the MGB definition of immunocompromising state have been published previously.^10^

5 The patient is only given this negative score if they are not immunocompromised.

6 Medications active against TB included: Rifampin, isoniazid, pyrazinamide, ethambutol, linezolid, tedizolid, amikacin, streptomycin, bedaquiline, clofazimine, pretomanid, rifabutin, rifapentine, levofloxacin, ciprofloxacin, and moxifloxacin.

During the Delphi process, infectious disease physicians provided insights on the consistency of the CDSS with clinical judgment. IPC staff provided guidance regarding elements of the patient’s history often lacking in the medical record at the time of individualized assessment to ensure collection of these details by the tool. MGB Digital Health information technology specialists contributed expertise around structured data collection and technical constraints.

### Improving usability of the initial model

Usability of the tool, focused on minimizing redundancy and maximizing clarity, was prioritized. Questions in the tool were reordered such that if the user indicated that there was no available chest radiology result at the time of CDSS activation, it would advise obtaining such imaging and returning to the tool without requiring the user to respond to the other questions. Branching logic was employed to display only questions that followed logically from previous responses; help text was added for clarity. For example, when asking about whether an individual’s symptoms have resolved or markedly improved with treatment for an alternative diagnosis and without any medications active against TB, a list of specific medications active against TB was added. Finally, the group reviewed the tool to ensure person-first language, formatting of epidemiologic questions to minimize stigma, and consideration of equity in the application of the tool to individuals from diverse backgrounds.

### Predictors of TB disease

Several predictors of TB infection were identified in the case–control analysis (Table 2). First, presence of a cavitary lesion or other findings specifically described as suspicious for TB on the radiology report was strongly associated with active TB as compared with the absence of these findings (chest radiograph [CXR]: odds ratio [OR]: 4.0; 95% confidence interval [CI]: 1.5, 11.5; chest computed tomography [CT]: OR: 3.3; 95% CI: 1.7, 6.2; *P* < 0.01). Prior residence in a highly TB endemic country (defined as anywhere outside of the United States, Canada, Western Europe, Australia, or New Zealand) was the strongest epidemiologic risk factor for TB identified (OR: 5.7; 95% CI: 2.6, 12.8; *P* < 0.01). Other traditional epidemiologic risk factors for TB were not significantly predictive, although our study was underpowered for such analysis (Table 2).

**Table 2.** Predictors of pulmonary TB infection in CDSS validation study

	Prevalence, n/N (%)		Unadjusted OR (95% CI)	
Predictor	Controls (Total = 104)	Cases (Total = 104)		P-value
**Chest Imaging**				
CXR: Cavitary lesion or findings described as suspicious for TB	7/92 (8%)	24/97 (25%)	4.0 (1.5, 11.5)	**<0.01**
CT chest: Cavitary lesion or findings described as suspicious for TB	32/96 (33%)	59/95 (62%)	3.3 (1.7, 6.2)	**<0.01**
**Epidemiology**				
Residence in a highly TB endemic country	44/83 (53%)	84/97 (87%)	5.7 (2.6, 12.8)	**<0.01**
Prior travel to a highly TB endemic country for >1 month (if no history of prior residence there)	5/34 (15%)	1/11 (9%)	0.6 (0.0, 6.2)	0.63
History of incarceration	8/38 (21%)	4/35 (11%)	0.5 (0.1, 2.1)	0.27
Prior residence in a homeless shelter	11/40 (28%)	3/28 (11%)	0.3 (0.1, 1.4)	0.09
Prior work in a correctional facility or homeless shelter	1/27 (4%)	2/26 (8%)	2.2 (0.1, 132.6)	0.53
Ever had known close contact with someone with active TB	9/55 (16%)	7/56 (13%)	0.7 (0.2, 2.4)	0.56
**TB History**				
Prior diagnosis of active TB	1/104 (1%)	5/104 (5%)	5.2 (0.6, 248.3)	0.10
History of latent TB	18/104 (17%)	24/104 (23%)	1.4 (0.7, 3.0)	0.30
Ever had a positive IGRA	17/104 (16%)	59/104 (57%)	6.7 (3.4, 13.7)	**<0.01**
Ever had a positive TST	13/104 (13%)	17/104 (16%)	1.4 (0.6, 3.3)	0.43
Negative IGRA or TST within the past 30 days	37/104 (36%)	15/104 (14%)	0.3 (0.1, 0.6)	**<0.01**
**Past Medical History**				
Immunocompromised	33/104 (32%)	20/104 (19%)	0.5 (0.3, 1.0)	**0.04**
Human Immunodeficiency Virus infection	10/104 (10%)	6/104 (6%)	0.6 (0.2, 1.8)	0.30
Underweight (body mass index <18.5)	7/104 (7%)	8/104 (8%)	1.2 (0.4, 3.9)	0.79
Diabetes mellitus	20/104 (19%)	17/104 (16%)	0.8 (0.4, 1.8)	0.59
Active head and neck cancer	5/104 (5%)	0/104 (0%)	0.0 (0, 0.74)	**0.02**
Chronic kidney disease	7/104 (7%)	3/104 (3%)	0.4 (0.1, 1.9)	0.19
Chronic obstructive pulmonary disease	9/104 (9%)	5/104 (5%)	0.5 (0.1, 1.9)	0.27
Active tobacco smoking	15/104 (14%)	8/104 (8%)	0.5 (0.2, 1.3)	0.12
Cirrhosis	2/104 (2%)	2/104 (2%)	1.0 (0.1, 14.0)	1.00
Gastrectomy or gastric bypass	0/104 (0%)	2/104 (2%)	n/a	0.16
None of the above	55/104 (53%)	63/104 (61%)	1.4 (0.8, 2.5)	0.26
**Symptoms**				
Cough	75/104 (72%)	63/104 (61%)	0.6 (0.3, 1.1)	0.08
Hoarseness	3/104 (3%)	5/104 (5%)	1.7 (0.3, 11.2)	0.47
Hemoptysis	21/104 (20%)	15/104 (14%)	0.7 (0.3, 1.5)	0.27
Fever	37/104 (36%)	48/104 (46%)	1.6 (0.9, 2.8)	0.12
Night sweats	21/104 (20%)	28/104 (27%)	1.5 (0.7, 2.9)	0.25
Weight loss	34/104 (33%)	55/104 (53%)	2.3 (1.3, 4.2)	**<0.01**
None of the above	16/104 (15%)	14/104 (13%)	0.9 (0.4, 2.0)	0.69
Symptoms resolved or markedly improved with treatment for an alternative diagnosis without TB treatment (asymptomatic individuals excluded)	38/88 (43%)	2/90 (2%)	0.0 (0.0, 0.1)	**<0.01**

OR: odds ratio; TB: TB, IGRA: interferon gamma release assay; TST: tuberculin skin test; CXR: Chest X-ray; CT: computed tomography.

Having a history of a prior diagnosis of active TB was uncommon among individuals in our study (controls: 1/104 [1%], cases: 5/104 [5%]), and we lacked power to detect a statistically significant difference in prior TB diagnosis (*P* = 0.10). History of latent TB was much more common (controls: 18/104 [17%], cases: 24/104 [23%]), but there was no statistically significant difference between groups (*P* = 0.30). Having a history of a positive IGRA result was a strong predictor of having active TB (OR: 6.7; 95% CI: 3.4, 13.7; *P* <0.01). Although IGRA and TST are not required for active TB evaluation, having a recent negative IGRA or TST within the past 30-days was negatively associated with TB (OR: 0.3; 95% CI: 0.1, 0.6; *P* <0.01). However, 14 of 104 individuals (14%) with culture-confirmed TB had a negative IGRA or TST within the 30 days prior to their TB diagnosis.

When assessed both individually and collectively, having a past medical history of conditions considered to increase the risk for TB were not associated with an increased odds of TB in our study.^7^ Immunocompromised status was more common among controls than among cases (OR: 0.5; 95% CI: 0.3, 1.0; *P* = 0.04), as was active head and neck cancer (OR: 0.0; 0.0, 0.74; *P* = 0.02). Similarly, except for a history of weight loss (OR: 2.3; 95% CI: 1.3, 4.2; *P* <0.01), no traditional TB symptoms were found to be predictors of active TB. Resolution or marked improvement of symptoms with treatment for an alternative diagnosis was strongly negatively associated with TB (OR: 0.0; 95% CI: 0.0, 0.1; *P* < 0.01).

### Model refinement and performance

**Figure 2.**
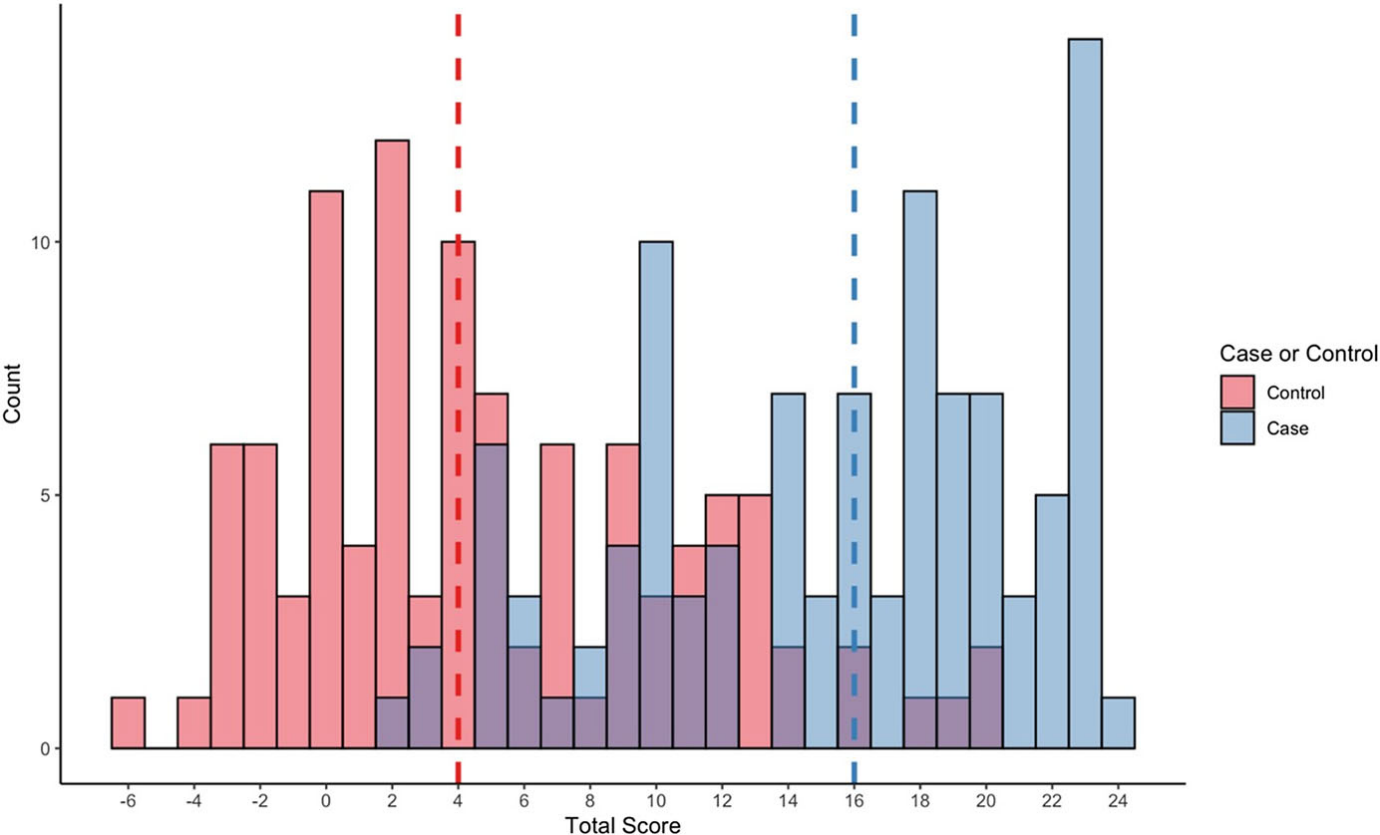
Distribution of scores between cases and controls in the final model. A histogram demonstrating the counts of total scores for controls (in red) and cases (in blue) is shown. Scores for controls ranged from −6 to 20. Scores for cases ranged from 2 to 24. The median values for total scores for controls (in red dashed lines) and cases (in blue dashed lines) are shown. Areas shaded in purple reflect overlap between the distributions of total score for cases and controls.

The initial model as designed through Delphi consensus demonstrated 100% sensitivity and 16% specificity with an AUC of 0.83. We made iterative revisions to the scoring system based on the results of the case–control study. First, given the lack of association between past medical history and active TB in our study, points for past medical history were removed; these questions were still preserved to aid in individualized review by IPC staff. Given the strong associations with the presence of active TB, scores for prior residence in a TB endemic country, weight loss, and substantial improvement in TB symptoms with treatment for an alternative diagnosis were upweighted. Questions regarding non-residence epidemiologic risk factors were downweighted. The overall subsection maximum score for epidemiology risk factors was also increased. Scores were higher for cases (median: 16, interquartile range [IQR]: 10, 20) than for controls (median: 4, IQR: 0, 9) in the final model (*P* < 0.01; Figure 2). The final model demonstrated 100% sensitivity and 27% specificity with an AUC of 0.87. Model performance was similar pre-COVID (*ie*, 100% sensitivity, 31% specificity, and AUC 0.93) and post-COVID (*ie*, 100% sensitivity, 23% specificity, and AUC 0.82).

### Coding and implementation

**Figure 3.**
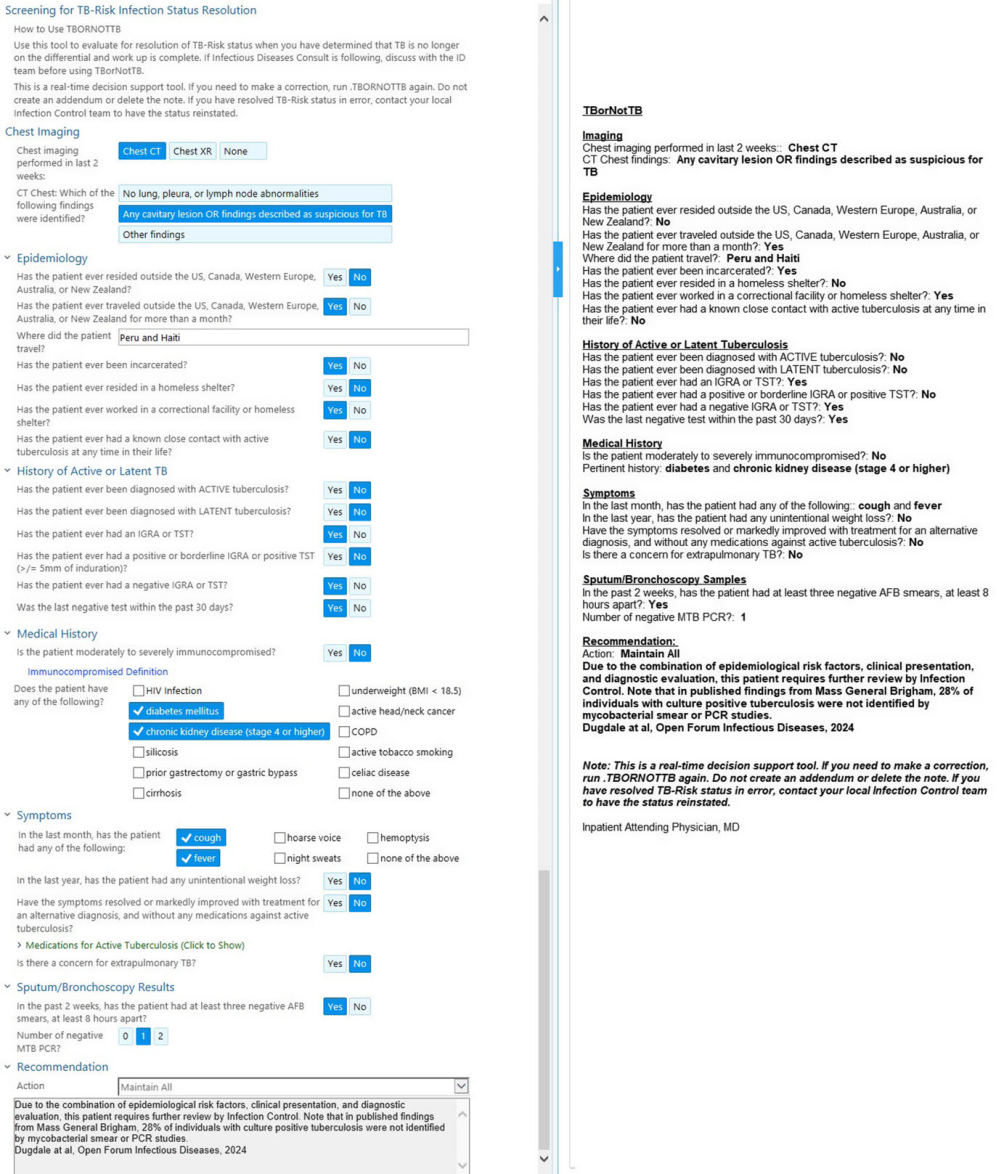
Screenshot of TBorNotTB CDSS in the Epic electronic medical record. From the top left, TBorNotTB guides clinicians to enter relevant recent radiology results. If no chest imaging is available (computed tomography or radiography), the clinician cannot proceed with using the tool and is advised of the requirement for radiological assessment. If chest imaging is available, the clinician can advance to document relevant epidemiology, followed by history of active or latent TB, then past medical history, symptoms, and finally sputum/bronchoscopy results. Using branching logic, questions are either hidden or appear based on the answers provided (*eg* if the answer is “No” to “Has the patient ever had an IGRA or TST,” the subsequent questions about results will not be revealed). Though not visible to the user of the tool, a weighted risk score is calculated in the background. If a final weighted score is below the established threshold, the TBorNotTB CDSS notifies the clinician using the tool that they can remove airborne infection isolation (AII). The tool also automatically removes the “TB-Risk” identifier on the patient’s chart upon signing the note. If, however, the risk score is above the threshold, TBorNotTB provides local site contact information for infection control to review the case further. If there is missing testing, TBorNotTB may also suggest further testing. Example screenshot from Epic™ (© 2024 Epic Systems Corporation). Video demonstration (mp4) of two patient scenarios using TBorNotTB CDSS is available in supplemental material, accessible at journals.cambridge.org.

A SmartForm-based tool (*ie*, advanced note template) was developed in the electronic health record Epic™ (Epic Systems Corporation, Verona, WI; Figure 3). During the build phase, the tool was accessed in a support environment (*ie*, an environment that contains patient data but does not write to the medical record) by the team of infectious disease and IPC experts to evaluate the CDSS usability with a variety of test cases. Based on these tests, further refinements were made to optimize frontline clinician understanding and user experience. In the live version of the tool, when the clinician completes the SmartForm, a note containing the information entered is automatically populated into the patient’s chart.

### Projected impact on IPC person-time

We estimated that use of the CDSS would replace >80 manual reviews conducted by IPC personnel each year, saving >40 IPC person-hours of time annually at MGH.

## Discussion

We developed and validated a novel real-time CDSS (TBorNotTB) to guide clinicians through the diagnostic evaluation of hospitalized individuals with suspected pulmonary TB in low-prevalence settings. We found that prior residence in a TB endemic country, positive IGRA, weight loss, continuation of symptoms despite treatment for alternative diagnoses, and findings concerning for TB on chest imaging were significant predictors of TB. CDSS contents and scoring were iteratively refined based on Delphi consensus and the case–control analysis. The revised CDSS demonstrated 100% sensitivity and 27% specificity to detect TB among hospitalized patients in a low-prevalence setting.

TB incidence in the US is on the rise. Concurrently, many hospitals across the US are experiencing capacity crises, making the expeditious evaluation of persons with possible infectious pulmonary TB even more urgent to preserve limited AII rooms. To provide guideline-recommended evaluation, optimize facility resources, and mitigate risk of nosocomial transmission, it is essential to determine when TB has been excluded with a high degree of confidence. AFB smears of respiratory specimens have limited sensitivity, even when multiple samples are submitted. NAATs improve sensitivity, but some individuals with culture-positive TB still have negative NAATs. Discontinuation of AII for TB often requires review by IPC personnel, but many facilities do not have adequate IPC personnel.^13,14^ Individualized review of patients with suspected TB, as well as contact tracing in the setting of nosocomial exposures, are very time- and resource-intensive endeavors for an already overstretched IP workforce.^13–15^

CDSS like TBorNotTB can support infection control activities by serving a range of functions, including packaging data for public health reporting, swiftly isolating and efficiently deisolating patients, triaging patients for testing and/or prophylactic treatment, and reducing cognitive and documentation burden for frontline clinicians. CDSS for infection control can also optimize hospital bed use and other resources. CDSS tools have been developed and deployed to support timely and appropriate deisolation for COVID-19 and suspected COVID-19,^6,16^ to support test-based clearance for MRSA and VRE,^17^ to screen patients for Ebola virus disease based on travel history and public health advisory,^18^ and to evaluate and recommend isolation, testing, and prophylactic treatment for patients with suspected mpox.^19,20^ Unlike paper-based tools, digital EHR-based tools to facilitate guideline-based infection control practice can be updated in real time to align with evolving science and policy, including in the context of emerging infectious diseases.

Several clinical prediction rules and scores are already in use to improve the detection of TB in a range of contexts and populations. Risk scores have been developed, for example, to stratify risk for adult contacts of index TB cases in Peru and for TB in smokers in Malaysia.^21,22^ Clinical risk prediction models have also been developed to predict pulmonary TB among individuals with suspected TB in Ethiopia, to predict bacteriologically confirmed TB in adults receiving antiretroviral therapy for HIV in Ethiopia, and to improve specificity of TB diagnosis among children living with HIV in Vietnam, Cameroon, and South Africa.^23–25^ While many other clinical decision support tools have been developed to direct TB treatment and testing, the TBorNotTB tool is the first to be leveraged to support IPC within healthcare settings.

The tools developed in our study and others highlight that CDSS is a powerful and flexible approach to address changing circumstances and novel science and technology. CDSS can be adapted to address different aspects of care in a standardized way, incorporating new epidemiological or other data, as well as advancements in laboratory methodologies over time. Healthcare facilities can adapt such tools to their local procedures or laboratory capacity, such as incorporating different testing protocols for evaluating suspected TB; some facilities require three negative AFB smears prior to removal of isolation precautions whereas others rely more heavily on NAAT-based testing.^7,10,26^ Another strength of CDSS is that the tool itself can collect and organize data that can be analyzed for future updates to structure or scoring to facilitate quality improvement.

Our analysis has several limitations. First, the CDSS test characteristics were assessed based on data from a single healthcare system in a high-income, low-prevalence setting. Epidemiologic risk factors and clinical characteristics among individuals with TB may differ in other low-prevalence cities or in high-prevalence settings, which could alter the performance of the tool. For example, in our setting some chronic medical conditions previously seen to increase the risk for TB disease such as diabetes and cirrhosis were not associated with TB in our population.^27,28^ These findings likely reflect enrichment for the presence of medical conditions in the control group that would lead clinicians to pursue three diagnostic AFB smears despite low to moderate pretest probability for TB. In these situations, evaluation for TB may have been pursued given the diagnostic uncertainties related to and clinical consequences of respiratory infection in the setting of immunocompromise or underlying lung disease, for example. Second, the CDSS validation dataset was derived from retrospective chart review, so there were some instances in which the EHR lacked sufficient detail to answer all questions. To address this, prospective assessment of the post-implementation performance of the CDSS, including its influence on duration of patient isolation, is planned. Third, the CDSS was only validated for use among hospitalized individuals undergoing evaluation for TB and not for ambulatory persons, although about half of all patients diagnosed with TB in the US have an initial hospitalization.^29^ Future analyses assessing application in the ambulatory population are needed.

In conclusion, we developed a clinical decision support system for the evaluation of suspected TB in a low-prevalence setting that demonstrated excellent sensitivity and modest specificity. This TBorNotTB CDSS is unique in its focus and application. Embedded into the electronic medical record system, this CDSS could help reduce both the risks of nosocomial TB transmission and the IPC person-time spent reviewing individuals with suspected TB.

## Supporting information

Dugdale et al. supplementary material Dugdale et al. supplementary material

## Supplementary material

The supplementary material for this article can be found at https://doi.org/10.1017/ice.2024.214
